# Spatial panorama of malaria prevalence in Africa under climate change and interventions scenarios

**DOI:** 10.1186/s12942-018-0122-3

**Published:** 2018-01-16

**Authors:** Francois M. Moukam Kakmeni, Ritter Y. A. Guimapi, Frank T. Ndjomatchoua, Sansoa A. Pedro, James Mutunga, Henri E. Z. Tonnang

**Affiliations:** 1 Human Health Division, International Center of Insect Physiology and Ecology, P.O. Box 30772-00100, Nairobi, Kenya; 2 Complex Systems and Theoretical Biology Group, Laboratory of Research on Advanced Materials and Nonlinear Science (LaRAMaNS), Department of Physics, Faculty of Science, University of Buea, P.O. Box 63, Buea, Cameroon; 3 Department of Computing, School of Computing and Information Technology, Jomo Kenyatta University of Agriculture and Technology (JKUAT), P.O. Box 62000-00200, Nairobi, Kenya; 4 Laboratoire de Mécanique, Département de Physique, Faculté des Sciences, Université de Yaoundé I, P.O. Box 812, Yaoundé, Cameroun; 5 International Maize and Wheat Improvement Center (CIMMYT) ICRAF House, United Nation, Avenue, Gigiri, Village Market, P.O. Box 1041, Nairobi, 00621 Kenya; 6 Departamento de Matemática e Informática, Universidade Eduardo Mondlane, Campus Principal, Maputo, Mozambique; 7 College of Biological and Physical Sciences, Institute for Climate Change and Adaptation (ICCA), University of Nairobi, P.O. Box 29053, Nairobi, Kenya; 8 School of Pure and Applied Sciences, Department of Biological Sciences, Mount Kenya University, P.O. Box 342-01000, General Kago Rd, Thika, Kenya

**Keywords:** Vector-borne disease, Transmission, Network model, Basic reproduction number, Geographical information system (GIS)

## Abstract

**Background:**

Malaria is highly sensitive to climatic variables and is strongly influenced by the presence of vectors in a region that further contribute to parasite development and sustained disease transmission. Mathematical analysis of malaria transmission through the use and application of the value of the basic reproduction number (*R*_0_) threshold is an important and useful tool for the understanding of disease patterns.

**Methods:**

Temperature dependence aspect of *R*_0_ obtained from dynamical mathematical network model was used to derive the spatial distribution maps for malaria transmission under different climatic and intervention scenarios. Model validation was conducted using MARA map and the Annual *Plasmodium falciparum* Entomological Inoculation Rates for Africa.

**Results:**

The inclusion of the coupling between patches in dynamical model seems to have no effects on the estimate of the optimal temperature (about 25 °C) for malaria transmission. In patches environment, we were able to establish a threshold value (about *α* = 5) representing the ratio between the migration rates from one patch to another that has no effect on the magnitude of *R*_0_. Such findings allow us to limit the production of the spatial distribution map of *R*_0_ to a single patch model. Future projections using temperature changes indicated a shift in malaria transmission areas towards the southern and northern areas of Africa and the application of the interventions scenario yielded a considerable reduction in transmission within malaria endemic areas of the continent.

**Conclusions:**

The approach employed here is a sole study that defined the limits of contemporary malaria transmission, using *R*_0_ derived from a dynamical mathematical model. It has offered a unique prospect for measuring the impacts of interventions through simple manipulation of model parameters. Projections at scale provide options to visualize and query the results, when linked to the human population could potentially deliver adequate highlight on the number of individuals at risk of malaria infection across Africa. The findings provide a reasonable basis for understanding the fundamental effects of malaria control and could contribute towards disease elimination, which is considered as a challenge especially in the context of climate change.

## Background

Malaria is one of the oldest and deadliest human vector-borne diseases. It is well known that malaria is transmitted among humans by the female Anopheles mosquito species, and the transmission cycle is essentially driven by the human-biting habit of the mosquito [1]. Malaria vectors are found in tropical and subtropical areas of the world and in sub-Sahara Africa, this disease is one of the major problems of public health [2, 3]. In 2015 an estimated 212 million (range 148–304 million) cases of malaria occurred worldwide and 429,000 people died, mostly children in Africa [2, 3]. In 2016, there were an estimated 216 million cases of malaria in 91 countries, an increase of nearly 5 million cases over 2015 [3]. The disease is endemic and/or epidemic, depending on the climatic parameters and ecological characteristics of the geographic regions [2, 3]. In general, occurrence of malaria vectors is organized into various patterns under the influence of temperature and the presence of breeding sites, which facilitates their reproduction and the increase of vector populations [2, 3]. These parameters therefore influence the epidemiology of malaria in a particular region.

The use of dynamic mathematical models to describe the time evolution of epidemiological systems dates back to centuries [4, 5]. The time evolution of the interactions between human population and malaria vectors are modeled using several methods, which include differential equations, discrete time maps, meta-population networks and geo-spatial approaches [6, 7]. Differential equations where space is ignored and the total number of individuals in the population of vectors or hosts is constant are mostly applied. In reality, the dynamics of populations are usually effected by a variety of interactions (both inter and intra); and as a consequence, it is appropriate to consider a grouping of small local population of vectors occupying small habitat zone “network” distributed in uniform matrix [8, 9]. In certain recent models of spreading epidemic, the location of the patches in space is treated explicitly by taking into account the number of connections *k* (degree) that any given patch within the network may have [10]. A common feature in the majority of such studies is the inclusion of temperature as a climatic variable [11]. Temperature-dependent models have been abundantly developed and the effects of temperature on population dynamics of malaria vectors have been well analyzed [12–14]. The proliferation of such modeling approach was motivated by the fact that temperature is a key element in the development of malaria vectors and parasites. A simulation model that includes the four life stages of mosquito life cycle (egg, larva, pupa, and adult) using delayed differential equations is presented in Depinay et al. [12]. Through, this model, the authors showed that the ambient temperature conditions play important roles both on the mosquito life-history processes and the parasites. In the study carried out by Mordecai et al. [13] temperature was introduced on the basic reproduction number (*R*_0_) and the results showed that the optimal temperature for malaria transmission is about 25 °C, whereas the work done by Parham and colleagues [14] estimated identical variable with a magnitude of 31 °C that is 6 °C higher than predicted in Mordecai et al. [13].

Mathematical analysis of the threshold values of *R*_0_ is an important and useful tool for the understanding of disease patterns. The basic reproduction number *R*_0_ depends on the following variables: mosquito density and biting rate, vector competence and survival rate as well as parasite development time within the mosquito (i.e. extrinsic incubation time) and human recovery rate, which are related to mosquito abundance, biology or physiology, and are linked to environmental conditions [13]. Mathematically, *R*_0_ is employed to characterize the possibility of a disease outbreak to occur if it exceeds 1 (*R*_0_ > 1), and the possibility of the disease to die out when *R*_0_ < 1. The quantity *R*_0_ can also help in determining the initial exponential increase in the number of infection during the disease outbreak [15]. In regions with endemic vector-borne diseases, it possible to determine potential control actions by varying the magnitude of the threshold value of *R*_0_ (usually 1). Such analysis provides appropriate guidance in the context of public health initiatives directed towards the reduction of the disease burden [14].

The control and eradication of infectious diseases are among the most important goals for improving public health. Although global eradication of communicable diseases (e.g. smallpox) was achieved [16], it has been difficult to eradicate malaria and other vector-borne diseases. Several countries in Africa, through diverse intervention measures [usage of artemisinin-combination therapy (ACT), long lasting insecticide-treated nets (LLINs), indoor residual spraying (IRS), mass screening and treatment (MSAT), etc.] have strengthened malaria control programs with perceptible triumph in decreasing both the disease incidence and sustained transmission. However, climate variability and changes associated to other factors are potentially redefining the conditions and areas of suitability and competence of malaria vectors and the risk of the disease as well as the sustenance of residual transmission [17, 18]. The most direct way in which climate change is projected to effect malaria is associated with the variation of ambient temperature. This climate variable has an impact on both the mosquitoes’ life-history processes and the parasites development; which both contribute to malaria transmission [13].

Geospatial science is currently offering new opportunities to infer spatial and temporal knowledge linking models that incorporate important disease characteristic patterns to modeling and mapping [19]. The outcomes of such models often yield potential zones of disease distribution and vector occurrence [19]. In this context, different modeling techniques have been used in mapping the spatial distribution of malaria vectors and transmission areas within local, regional and global scales [20–25]. In [26] advanced statistical techniques (non-linear discriminant analysis, random forest, and generalized linear model) were employed to investigate the environmental suitability in the Netherlands for three indigenous mosquito species. In [27] the annual entomological inoculation rate for the *Plasmodium falciparum* transmitted by *Anopheles gambiae* was utilized as a proxy to map the intensity of malaria transmission in Uganda. Climate parameters coupled with areas of seasonal abundance were used for spatial simulations of key malaria vectors (*A*. *gambiae* and *Anopheles arabiensis*) in Africa [28, 29]. Justifications in undertaking these studies were to reduce the disease burden through knowledge and information dissemination to empower public health workers and policymakers in better approaches to management of malaria. Still in this direction, the present study uses temperature-dependence aspect of the reproductive number obtained from dynamical mathematical network model to derive spatial distribution maps under different climatic and intervention scenarios to understand and forecast malaria transmission dynamics. The roles played by different interventions to control the disease and changes in temperature on the basic reproduction number are well analyzed.

## Methods

### Temperature data

The spatial simulation of *R*_0_ is made with temperature data obtained from WorldClim (http://www.worldclim.org/) and CCAFS (Climate Change, Agriculture and Food Security; http://www.ccafs-climate.org) databases. Data for the year 2000 were used to analyze the present situation whereas simulated data for the year 2050 were used to represent future scenario. The data are layers (grids) with a spatial resolution of 2.5 min containing average minimum and maximum temperatures.

### Mathematical model

Among existing mathematical models developed for malaria, the model of Ross and Macdonald [30], which has laid the foundation of majority of current epidemiological models has attracted a lot of considerations. In conducting the spatial panorama of malaria prevalence in Africa under climate change and interventions scenarios, the Ross and Macdonald model [30] was selected. This model is entrenched on the assumption that at any given moment, an entire population of either humans or vectors can be divided into distinct compartments made of the susceptible (those who are vulnerable to infection) and infectious (those that have acquired infection and are able to infect others). The infection spreads by random contact between susceptible and infectious “compartments” of the human and mosquito populations [31]. The Ross–Macdonald model has two equations for describing the changes in the number of infectious hosts and vectors. The changes in the number of susceptible hosts and vectors are implicitly modeled since the host and vector population sizes are kept constant [32]. The patchy model of the system was proposed in [33] by considering vectorial transmission. Such transmission is considered to be strongly correlated to climate variables, which heavily influence the distribution of vectors across geographical scales [11]. However, the extent to which current and future projections of temperature contribute to the patterns of disease prevalence is yet to be well established. This understanding is of great importance in evaluating and better preparing for future malaria transmission/risk, and would inform strategies of addressing current residual malaria transmission in Africa. A multi-malaria model inter-comparison that considered the impact of climate change on the disease transmission at global scale is presented in [18]. Herein, we use a patchy process-based model that accounts for both the vector and parasite influences. From the model, the basis basic reproduction number *R*_0_ was derived and used to project the disease transmission at scale. Then the result was reproduced at country level to both analyze climate change and intervention impacts on the population. The latter factor, which is often neglected in most studies was considered here as the element that brings originality into the present research. In this framework, the Ross–MacDonald model in patch environment is described by the following equations [33].

1 $$ \left\{ {\begin{array}{l} {\frac{{du_{i} }}{dt} = apv_{i} \frac{{M_{{H_{i} }} - u_{i} }}{{M_{{H_{i} }} }} - \xi u_{i} + \mathop \sum \limits_{j = 1, j \ne i}^{n} m_{ij} u_{j} - u_{i} \left( {\mathop \sum \limits_{j = 1, j \ne i}^{n} m_{ij} } \right)} \\ {\frac{{dv_{i} }}{dt} = aq\left( {M_{{V_{i} }} - v_{i} } \right)\frac{{u_{i} }}{{M_{{H_{i} }} }} - \delta v_{i} } \\ \end{array} } \right. $$

The variables *u*_*i*_ and *v*_*i*_ are the numbers of infected host and vector respectively, in each patch. Without the loss of generality, the following variables were kept constant in all patches and are defined as: *a* is the biting rate of the vector, *p* is the probability that the bite of infectious vector will lead to the successful infection of a susceptible host, *q* is the probability that a susceptible vector that bites an infectious host will become infected, *ξ* is the rate at which infectious hosts recover, *δ* is the mortality rate of infected vectors and, *M*_*Hi*_ represents the total host population in patch *i*, and *M*_*Vi*_ is the vector population in patch *I*; *m*_*ij*_ is the migration rate from patch *i* to patch *j* with *i* ≠ *j*.

### Basic reproduction number in patches environment

The basic reproduction number generally represented by *R*_0_ is a fundamental quantity in the study and analysis of mathematical models in epidemiology. *R*_0_ is commonly defined as the number of secondary cases that can be produced by one case of an infected individual in a completely susceptible population. The general framework for the determination of the basic reproduction number can be found in [15]. *R*_0_ is presented for different disease transmission models based on a system of differential equations. The expression of $R_{0}$ for the patchy Ross–Macdonald model presented above is given in [33]. To observe the effect of the coupling model components, we sequentially analyzed the model with single and double patches. The expression of the basic reproduction number for a single patch is given by [32]:

2 $$ \hat{R}_{0}^{2} = a^{2} \frac{pq}{\delta \xi }*\frac{{M_{V} }}{{M_{H} }} $$

With double patches, the expression of the basic reproduction number is more complex and is given as follows [33]:

3 $$ \begin{aligned} R_{0}^{2} & = a^{2} \frac{pq}{{\delta \xi M_{H} }}\frac{{\left( {m_{12} + m_{21} } \right)}}{{2\left( {\xi + m_{12} + m_{21} } \right)}}\left[ {\left( {1 + \frac{\xi }{{m_{12} }}} \right)M_{{V_{1} }} + \left( {1 + \frac{\xi }{{m_{21} }}} \right)M_{{V_{2} }} } \right. \\ & \quad \left. { +\, \sqrt {\left( {\left( {1 + \frac{\xi }{{m_{12} }}} \right)M_{{V_{1} }} + \left( {1 + \frac{\xi }{{m_{21} }}} \right)M_{{V_{2} }} } \right)^{2} + 4M_{{V_{1} }} M_{{V_{2} }} } } \right] \\ \end{aligned} $$

The *m*_12_ and *m*_21_ are the migration rates from patch 1 to patch 2 and from patch 2 to patch 1 respectively, $M_{{V_{1} }}$ and $M_{{V_{2} }}$ are the vector population densities in patch 1 and 2 respectively, while *M*_*H*_ is the total host population per patch.

Two cases were considered in the analysis:

1. If the population is distributed uniformly, then the migration parameters are set to *m*_12_ = *m*_21_ = *k*. In this instance, the identical proportion of vector population migrates from one patch to another, while keeping the overall populations at individual patches constant. Under this assumption the formulation of the basic reproduction number is given by:
  4 $$ R_{0}^{2} = 2\hat{R}_{0}^{2} $$
  5 $$ \begin{aligned} R_{0}^{2} & = a^{2} \frac{pq}{{\delta \xi M_{H} }}\frac{2k}{{2\left( {\xi + 2k} \right)}}\left[ {\left( {1 + \frac{\xi }{k}} \right)M_{{V_{1} }} + \left( {1 + \frac{\xi }{k}} \right)M_{{V_{2} }} } \right. \\ & \quad \left. {  +\,\sqrt {\left( {\left( {1 + \frac{\xi }{k}} \right) (M_{{V_{1} }} + M_{{V_{2} }} )} \right)^{2} + 4M_{{V_{1} }} M_{{V_{2} }} } } \right] \\ \end{aligned} $$
2. Here, the population is distributed uniformly, however, the migration parameters are different (*m*_12_ ≠ *m*_21_) and the population of infected vectors in one of the patches is set to 0. This case depicts a situation whereby the population of infectious mosquitoes in one patch is maximum and null in the other patch. With these considerations, the expression of the basic reproduction number, which depends on coupling and other parameters of the system, is given by:
  6 $$ R_{0}^{2} = a^{2} \frac{pq}{\delta \xi }*\frac{{M_{{V_{1} }} }}{{M_{H} }}\left( {1 + \frac{{\xi m_{21} }}{{m_{12} \left( {m_{12} + m_{21} + \xi } \right)}}} \right) $$

The existence of an *endemic equilibrium* in the model is guaranteed when $R_{0} > 1$ [34]. The control of vectors contribute to the decrease of vector-host ratio, which reduces the basic reproductive ratio of the pathogen; then decreases the equilibrium number of infectious hosts and vectors. Therefore, for successful disease control, the vector population density has to be reduced at least to a level below the *entomological threshold* that is mathematically expressed as:

7 $$ \frac{{M_{V} }}{{M_{H} }} > \frac{\delta \xi }{{a^{2} pq}} $$

### Parameterization of the reproductive number

To parameterize the reproductive number, we opted to undertake a literature search. In Eq. (3), there are 10 variables, among which five are found to be dependent on temperature. They have been well described by authors in [34]. Therefore, under the assumptions that vectors and parasites are dependent on temperature, the function to represent the reproductive number as temperature dependent for a single patch is given in Eq. (8) below:

8 $$ \hat{R}_{0}^{2} (T) = a^{2} (T)\frac{p\left( T \right)q(T)}{\delta (T)\xi }*\frac{{M_{V} (T)}}{{M_{H} }} $$

Similarly *R*_0_ for two patches is obtained by:

9 $$ \begin{aligned} R_{0}^{2} (T) & = a^{2} (T)\frac{p\left( T \right)q(T)}{{\delta (T)\xi M_{H} }}\frac{{\left( {m_{12} + m_{21} } \right)}}{{2\left( {\xi + m_{12} + m_{21} } \right)}}\left[ {\left( {1 + \frac{\xi }{{m_{12} }}} \right)M_{{V_{1} }} (T) + \left( {1 + \frac{\xi }{{m_{21} }}} \right)M_{{V_{2} }} (T)} \right. \\ & \quad \left. {  +\, \sqrt {\left( {\left( {1 + \frac{\xi }{{m_{12} }}} \right)M_{{V_{1} }} (T) + \left( {1 + \frac{\xi }{{m_{21} }}} \right)M_{{V_{2} }} (T)} \right)^{2} + 4M_{{V_{1} }} \left( T \right)M_{{V_{2} }} (T)} } \right] \\ \end{aligned} $$

In this Eq. (9), other parameters *ξ*, *m*_12_, *m*_21_, and *M*_*H*_ are kept constant. In [13], the optimal temperature for malaria transmission was estimated at around 25 °C. This estimate is close to the values obtained from a study on entomological inoculation rates [35]. Hence, for our parameterization we opted for the approach proposed by [13] in which a constant number of vector population density is assumed in each patch. The quantity *M*_*Vi*_(*T*) (*i* = 1,2) is estimated as:

10 $$ M_{{V_{i} }} (T) = \frac{{EFD_{i} \left( T \right)P_{{EA_{i} }} (T)}}{{\tau_{EAi} \left( T \right)\delta (T)}} $$

*EFD*_*i*_(*T*) is the number of eggs laid by a vector per day, *P*_*EAi*_(*T*) is the probability that an egg survives to become an adult mosquito vector, and *τ*_*EA*_*i*(*T*) is the duration of egg to adult development in each patch *i*. The relation $p\left( T \right)q(T) = \phi (T)e^{ - \delta \left( T \right)EIP(T)}$ expresses the product of the probability that the bite of an infectious vector will lead to the successful infection of a susceptible host *p*, and the probability that a susceptible vector that bites an infectious host will become infected *q* with the vector competence *ϕ*(*T*). The mortality rate is *δ*(*T*) and the extrinsic inoculation rate is *EIP*. Using relation (9) and replacing the parameter time by rate, the complete temperature dependent *R*_0_ for malaria can be described as per the following cases:

When the system has only one patch Eq. (2) is given by:

11 $$ \hat{R}_{0}^{2} \left( T \right) =     \frac{{a\left( T \right)^{2} \phi \left( T \right)e^{{\frac{ - \delta \left( T \right)}{PDR\left( T \right)}}} EFD\left( T \right)P_{EA} \left( T \right)MDR\left( T \right)}}{{M_{H} \xi \delta^{3} \left( T \right)}} $$

When the system possesses two patches, then two sub-cases are generated and their equations are:

1. $R_{0}^{2} (T) = 2\hat{R}_{0}^{2} (T)$ with $\hat{R}_{0}^{2} (T)$ defined by Eq. (10),
2. $$ R_{0}^{2} = a^{2} (T)\frac{{\phi (T)e^{ - \delta (T)/PDR(T)} }}{{\delta (T)^{3} \xi }}\frac{{EFD\left( T \right)P_{EA} \left( T \right)MDR(T)}}{{M_{H} }}\left( {1 + \frac{{\xi m_{21} }}{{m_{12} \left( {m_{12} + m_{21} + \xi } \right)}}} \right) $$

Table 1 is the summary of the temperature dependent expressions of *R*_0_ parameters. They were obtained from the analysis described in Mordecai et al. [13].

**Table 1:** Temperature dependent parameters [13]

Variables	Definition	Mathematical expression	Estimate of parameters
*a*	Biting rate	*cT*(*T* − *T*_0_) (*T*_*m*_ − *T*)^1*/*2^	*c* = 0.000203; *T*_*m*_ = 42.3; *T*_0_ = 11.7
*ϕ*	Vector competence	*qT*^2^ + *rT* + *s*	*q* = − 0.54; *r* = 25.2; *s* = − 206
*δ*	Adult mortality rate	[*ln*(*qT*^2^ + *rT* + *s*)]^−1^	*q* = − 0.000828; *r* = 0.0367; *s* = 0.522
*PDR*	Parasite development rate	*cT*(*T* − *T*_0_) (*T*_*m*_ − *T*)^1*/*2^	*c* = 0.000111; *T*_*m*_ = 34.4; *T*_0_ = 14.7
*EFD*	Eggs laid per adult female per day	*qT*^2^ + *rT* + *s*	*q* = − 0.153; *r* = 8.61; *s* = − 97.7
*P*_*EA*_	Egg-to-adult survival probability	*qT*^2^ + *rT* + *s*	*q* = − 0.00924; *r* = 0.453; *s* = − 4.77
*MDR*	Mosquito development rate	*cT*(*T* − *T*_0_) (*T*_*m*_ − *T*)^1*/*2^	*C* = 0.000111; *T*_*m*_ = 34; *T*_0_ = 14.7

More details about the derivation and errors calculations can also be found in supplementary materials [13]

### Spatial projection of the reproductive number

Many countries in Africa experience a spatial variation of temperatures and the predictions of humidity and precipitation are highly uncertain, here temperature was considered as a key factor. The selection of temperature alone is further justified by the fact that a small increase in the value of this variable leads to a significant increase in malaria vector development time and in the frequency of blood feeding in adults [36]. It has been well established that the parasites responsible for malaria occurrence are transmitted to their hosts during blood meals by female vectors [36]. Therefore, to regionally map *R*_0_, the monthly *max* and *min* temperatures are simultaneously extracted from the databases and their average estimated. The obtained information is organized into matrices with longitude as the column and latitude as the row. A point object, representing the temperature dependent mathematical expression of *R*_0_ is generated and applied in each geographical coordinate. A new matrix is formed with the values of the *R*_0_ in the respective geographical coordinates. The results are converted to American Standard Code for Information Interchange (ASCII) files and transferred to an open source software Q-GIS [37] for visualization of the potential risk areas of malaria transmission under selected climate scenarios. A similar approach was used for the intervention scenarios. Overall, the method exploits the spatial variation of temperature to provide geographical predictions.

### Climate change and intervention scenarios

The choice of scenarios was guided by the availability of datasets, which were obtained from WorldClim (http://www.worldclim.org/) and CCAFS (Climate Change, Agriculture and Food Security) (http://www.ccafs-climate.org) databases. Downscale datasets for the year 2050 (future climate) of the SRES-A1B [38] was chosen for the analysis.

To take into account the contribution of different interventions on the burden of malaria in Africa for our projections, we identified studies that recorded trends in the disease indicator over a period of time [39]. The study demonstrated that vector control has immensely contributed to the decline of the burden caused by malaria in sub-Saharan Africa. With the selected modeling framework, four parameters (mosquito biting rate *a*, vector competence *bc*, adult mosquito mortality rate *µ*, and the probability that mosquito eggs survive to become adult *P*_*EA*_), which are directly linked to malaria vectors population were adjusted based on intervention effects. The application of the interventions criteria on these parameters has a direct impact on the amplitude of the basic reproduction number *R*_0_. The following two intervention scenarios were adopted: (1) uniform application of 40% reduction in the amplitude of *a*, *bc*,*µ* and *P*_*EA*_ respectively; and (2) selection of two African countries and reducing the magnitude of *a*, *bc*,*µ* and *P*_*EA*_ by up to 40% (Cameroon) and 80% (Kenya) [40, 41]. The baseline for the beginning of interventions was set to year 2000. Year 2050 represents the future scenario to measure the impacts of the interventions. In the analysis, the temperature values of the years 2000 and 2050 were successively replaced by the variable *T* in the mathematical expression of the reproductive number *R*_0_.

### Model validation

The process of determining the degree to which the simulated regional maps are accurate was conducted through comparison of observed risk areas for malaria transmission as described in [17, 42] with the maps of the basics reproduction rate *R*_0_ obtained from the present analysis. The *P. falciparum* entomological inoculation rate (*Pf*EIR) is a measure of the proportion of exposure to infectious mosquitoes [27]. It is often translated as the amount of *P*. *falciparum* infective bites received by each human within a season or annually [27]. The synthesis of Annual *P. falciparum* Entomological Inoculation Rates (*APfEIR*) data found in the literature of 22 spatially distinct records in Africa, post-1980, across the continent was used to compare the values of *R*_0_ obtained from our model [17, 42].

## Results

In the present context, the basic reproduction number *R*_0_ is used as a tool in evaluating and predicting risk zones for malaria transmission. High values of *R*_0_ (> 1) correspond to malaria risk zones. Our results indicate that for a population density of *M*_*H*_ = 100 and an immunity coefficient of *ξ* = 0.1, the values of *R*_0_ range between 0 and 9. Such values provide an overall view of the intensity of malaria transmission in the region. The areas in Africa where *R*_0_ was close to the value 9 correspond to locations with high malaria prevalence. The value of *R*_0_ tends to zero in areas with low risk of malaria transmission. The maximum values of *R*_0_ are obtained at the locations where the optimal temperature for malaria transmission is about 25 °C.

**Fig. 1:**
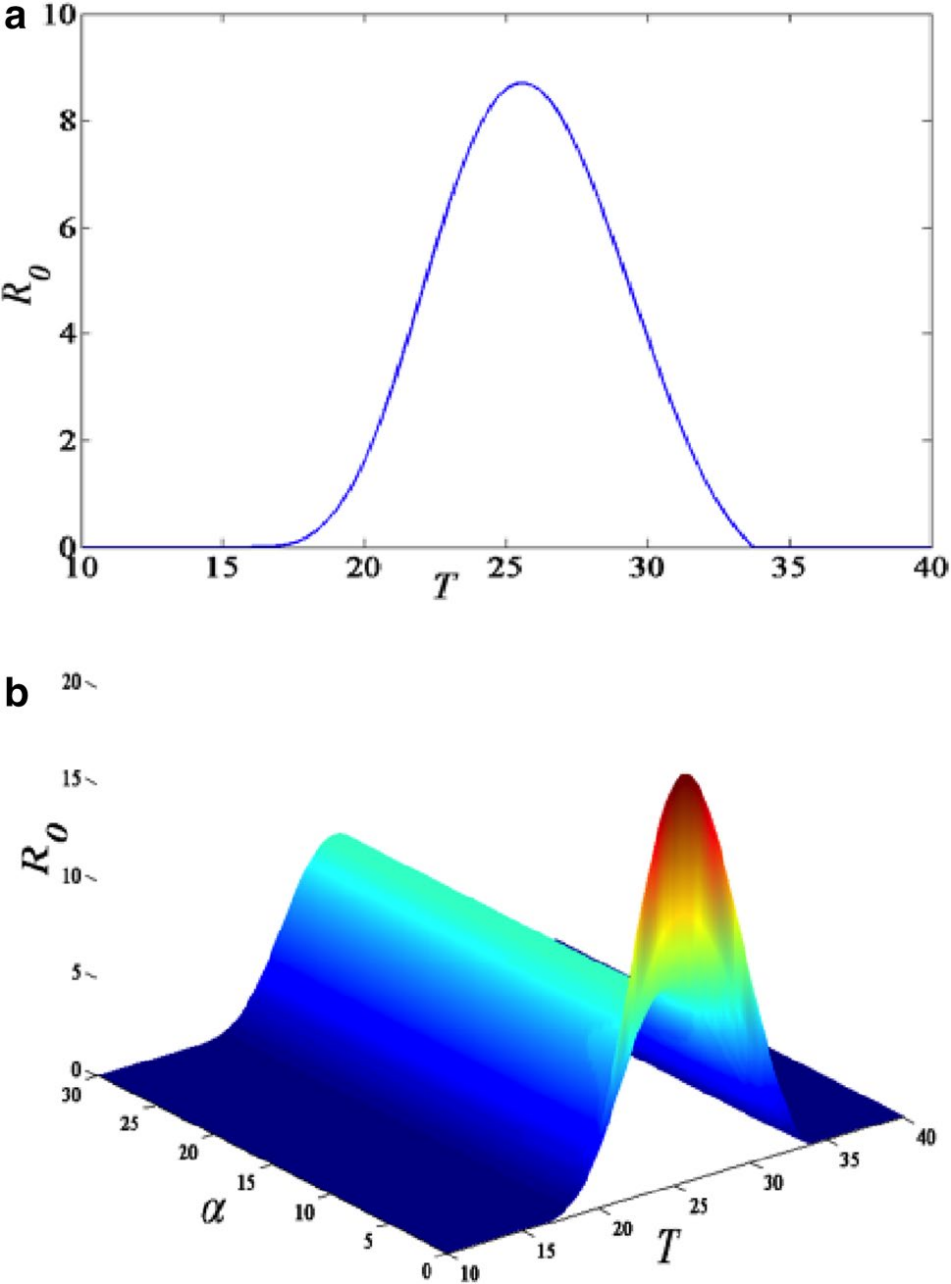
**a** Plot of the basic reproduction number *R*_0_ expressed as a function of temperature and **b** 3D plot of the temperature-dependent basic reproduction number *R*_0_ for two patches as a function of the migration ratio between the patches

In the case of one patch, the graph of *R*_0_ is represented in Fig. 1a. For two patches, if *m*_12_ = *m*_21_, the value of the basic reproduction number doubles. When *m*_12_ ≠ *m*_21_, we set *m*_12_ = *α m*_21_ and substituted it in Eq. (3) and then-plotted *R*_0_ in three dimensions as presented in Fig. 1b. The result of the simulation was quite interesting as highlighted herein. In Fig. 1b, it was observed that as the ratio of the migration between the two patches increases, the basic reproduction number decreases up to a threshold value *α* = 5. Furthermore, an increase of the value of *α* does not change the value of *R*_0_. In this circumstance, the numerical value of *R*_0_ obtained when *α* > 5 (i.e. where the basic reproduction number converge) is identical to the value estimated by Ross–Macdonald model with a single patch. When*α* = 1, the basic reproduction number is the double of the critical value of *R*_0_ obtained at *α* > 5.

**Fig. 2:**
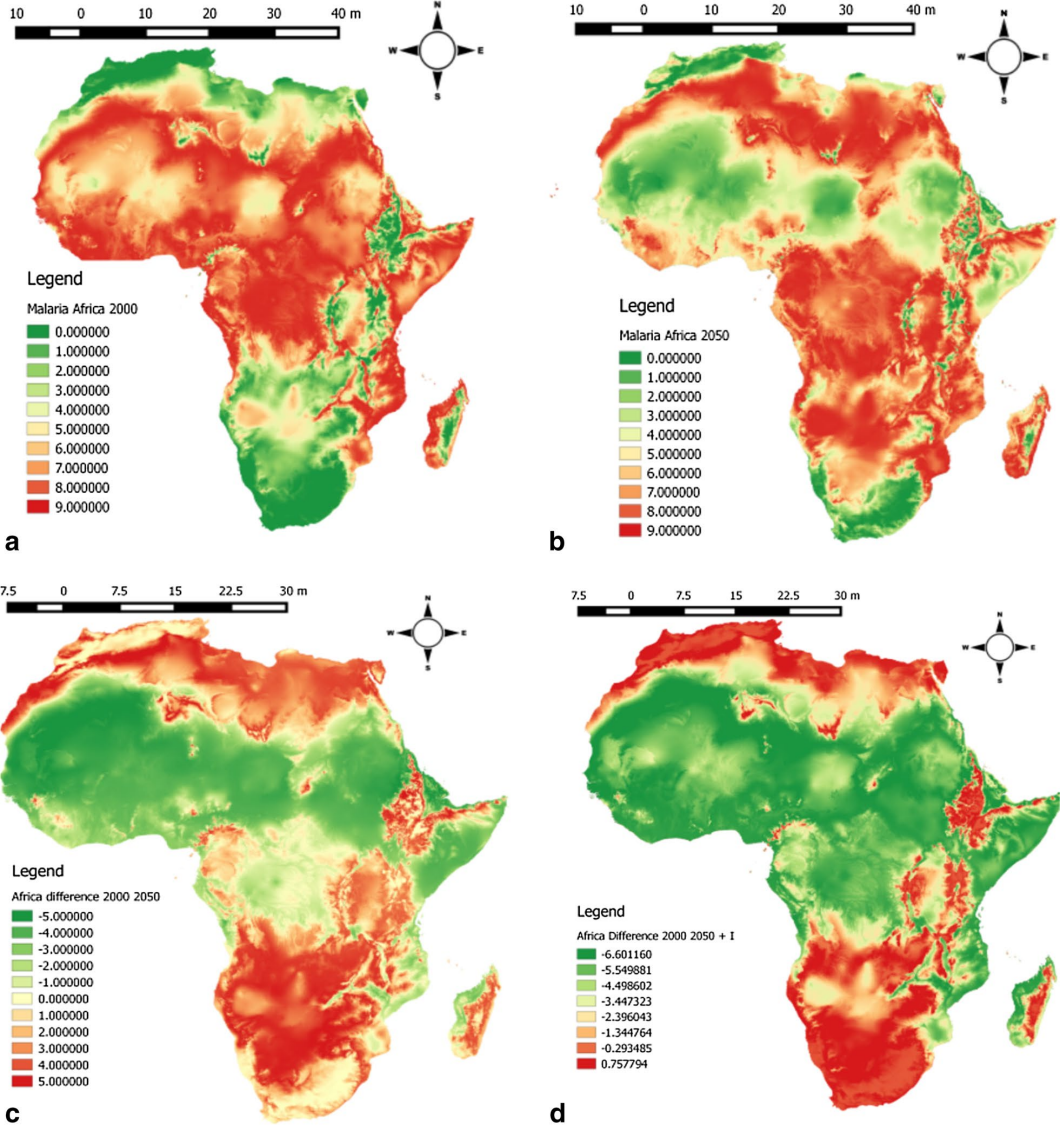
Maps illustrating the spatial distribution of malaria transmission using the basic reproduction number *R*_0_ derived from a mathematical model. **a** Distribution of the baseline scenario obtained by replacing the variable temperature with the values of the year 2000; **b** malaria transmission map for future scenario corresponding to substituting temperature with the values of the year 2050. **c**, **d** Differences on the values of *R*_0_ between the baseline (year 2000) and future scenario (2050) without (**c**) and with (**d**) interventions respectively. Without intervention means only the predicted values of temperatures are substituted into the expression of *R*_0_ while with interventions signify that in addition to replacing the values of temperatures with the predicted values of the year 2050, a changes in the values of four model parameters (per mosquito biting rate *a*, the vector competence *bc*, the adult mosquito mortality rate *µ*, and the probability that mosquito eggs survive to become adult *P*_*EA*_) were conducted

**Fig. 3:**
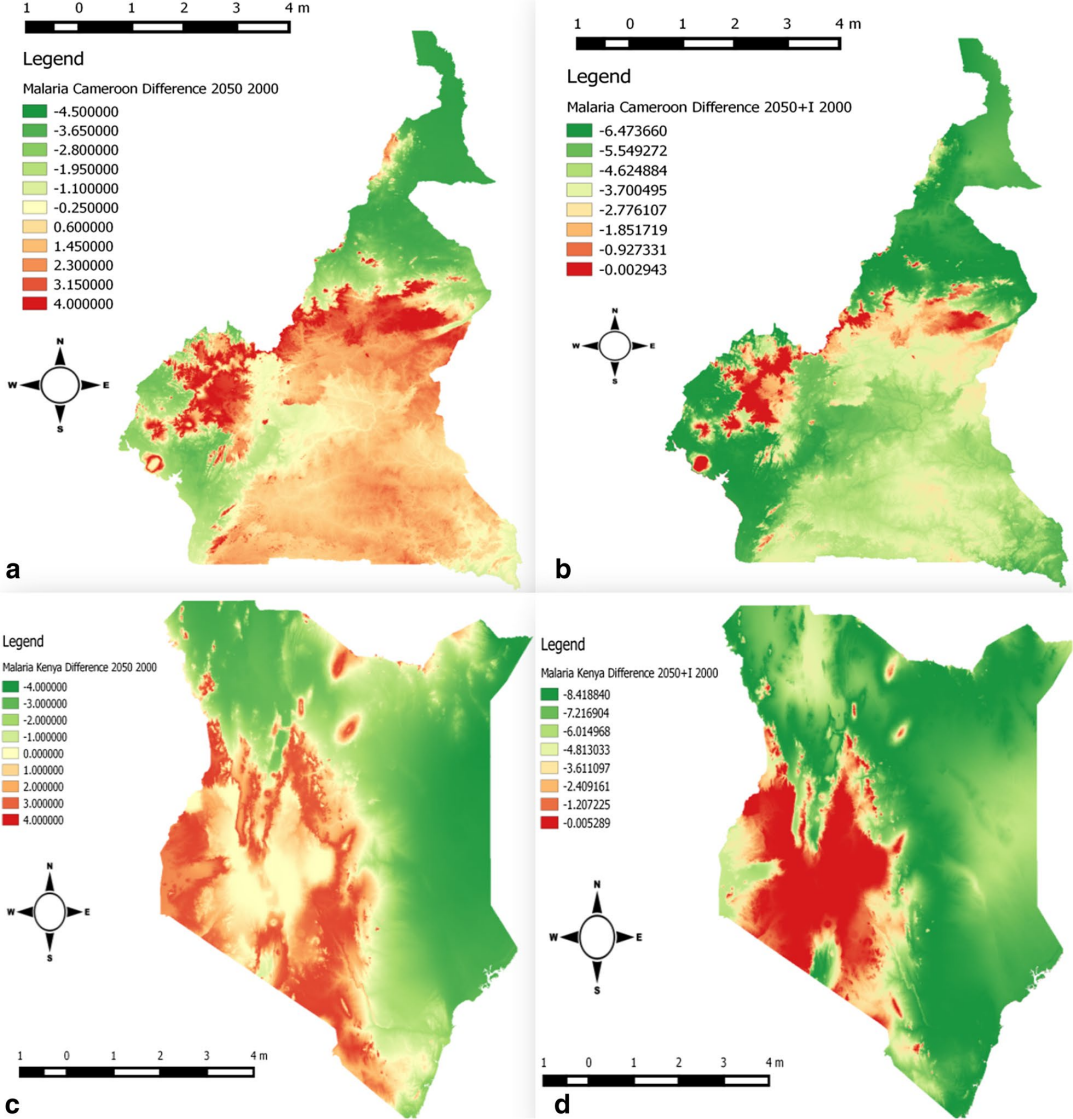
Zooming in at country level to illustrate the spatial distribution of malaria transmission inferred by the basic reproduction number *R*_0_. It shows the changes in the values of *R*_0_ computed from the subtraction of the baseline (year 2000) to future scenario (2050) without (**a**, **c**) and with (**b**, **d**) interventions for Cameroon and Kenya respectively. The interventions scenarios were deduced by reducing the magnitude of certain values of the model parameters by up to 40% (Cameroon) and 80% (Kenya) respectively

It was observed that the introduction of the coupling between the patches only has effects on the amplitude of *R*_0_ and not the optimal temperature for the transmission of malaria. In order words, a model with one or two patches will produce almost similar outcomes when projected spatially. Based on these results, we decided to limit the production of the spatial distribution map of the basic reproduction number *R*_0_ to a single patch model given by Eq. (11). The distribution maps predicting the values of *R*_0_ in Africa are shown in Figs. 2 and 3. The predictions comprehensively match the known distribution map for malaria transmission and endemic zones in Africa.

The map representing the present distribution (Fig. 2a) inferred by the degree of magnitude of *R*_0_ indicates greater values in most malaria endemic regions such as west and central Africa. The magnitude of *R*_0_ considerably reduced as we move northward and southward and became almost close to zero in the temperature regions of northern and southern parts of Africa, which are currently characterized by a very low level of malaria transmission. A similar trend is observed in the highlands of eastern Africa, South Africa, western Cameroon, central Angola and the plateau of Madagascar.

For future projection (2050), we observe a shift in areas of transmission of the disease towards the northern and southern regions of Africa (Fig. 2b). A projection of the developed model indicates a high risk of transmission and establishment of the disease in these regions. Malaria transmission will remain present in regions close to the equator. The future scenario also indicates that the level of malaria transmission in the Sahara Africa will highly reduce.

The changes in the values of *R*_0_ due to the temperature change between the years 2010 and 2050 are presented in Fig. 2c. The map displays the difference *ΔR*_0_ = *R*_01_ − *R*_02_, where *R*_01_, is the basic reproduction of the baseline scenario obtained by replacing the variable temperature with the values of the year 2000 and, *R*_02_ the malaria transmission map for future scenario corresponding to substituting temperature with the values of the year 2050. Two classes of singularities are observed on the map: (1) areas where there will be an increase in the value of *R*_0_, and (2) areas in which *R*_0_ values decrease considerably. The increases in the values are found in regions mores close to the equator and at the Horn of Africa. The southern and northern regions of Africa are also of great concern given the observed increase in value of *R*_0_ (Fig. 2c), which demonstrates a possibility of disease endemicity in these areas.

The effects of interventions were computed from the subtraction of the baseline (year 2000) map to future scenario (2050) map, in which changes in the amplitude of certain models parameters as described above were made. By including intervention measures into the model, most of the regions are likely to experience a decrease in the values of *R*_0_ (Fig. 2d). The central and eastern African highlands and the southern regions of Africa are with the least decline in *R*_0_ values. Current steady areas where malaria is endemic are characterized by values of *R*_0_ that tend to zero; justifying the efficiency of the interventions in reducing the disease burden.

The maps of two selected countries with different intervention levels are presented in Fig. 3. This analysis was carried out in order to observe the effect of the intervention at the local scale. For Cameroon, a large variation in the value of *R*_0_ is observed in western highlands and Adamawa Region (Fig. 3a, b). The magnitude of the difference *ΔR*_0_ varies from − 4.5 to 4. We defined, *ΔR*_0*I*_ = *R*_01_ − *R*_02*I*_ as the difference between the values of the reproduction number obtained for the baseline (the year 2000) and future scenario (the year 2050) with and without interventions; respectively. The application of the intervention measures considerably decreased the magnitude of *ΔR*_0*I*_ as many points on the map have negative values. These values range between − 6.4736 and − 0.0029. The smaller the value of *ΔR*_0*I*_, the higher the impact of the intervention(s).

The maps of Kenya (Fig. 3c, d) indicate the values of *ΔR*_0_ ranging between − 4.00 and 4.00. By applying the intervention measures, the changes in the areas of prevalence between 2000 and 2050 (*ΔR*_0*I*_) ranges from − 8.4188 to 0.0052. The lowest magnitude in the change of the values of the basic reproduction number was observed in highland areas of the countries. Overall, the changes in the values of *R*_0_ are more pronounced in Kenya than in Cameroon as Kenya may have benefited from high level of interventions than Cameroon.

To further confirm the performance of our approach, we overlaid the entomological *APfEIR* survey data with the magnitudes of the basic reproduction number. By considering a region with the *APfEIR* value greater than 0 as endemic, and if *APfEIR* < 1, the region is moderately endemic. *APfEIR* > 10 were predicted for areas with very high risk of the disease transmission. In Table 2 the summary of the basic reproduction numbers computed for sites specific to the values of *APfEIR* are presented. The values of the basic reproduction number are large enough in all sites corresponding to high values of *APfEIR*. Overall, the maps of *R*_0_ for malaria endemicity revealed minimal bias with predicted *APfEIR* map. Nevertheless, the present map with *R*_0_ has the general tendency to overestimate prevalence by *R*_0_. The matching between predicted map of *R*_0_ and the estimates of *APfEIR* map indicate consistent agreement in the 2 parameters.

**Table 2:** Summary for validation of the map comparing the APfEIR with the predicted value R_0_ by site [43]

Site	Latitude	Longitude	*APfEIR*	*R*_0_
Cotonou-Centre	6.35	2.43	39.06	8.28
Koubri	12.15	− 1.38	441.6	6.81
Gisenga	− 4.44	29.67	251.7	5.54
Mutengene, Molyko, Likoko, Vasingi	4.08	9.3	160	8.61
Kulila	− 4.17	12.43	397.9	7.39
Kinshasa, rural area	− 4.47	15.31	620.5	8.64
Alloukoukro	7.8	− 5.08	231.5	8.37
Abheet	29.42	30.83	1.8	3.19
Magdalena Mora	3.73	8.8	598.14	8.08
Franceville, Akou suburb	− 1.63	13.45	81.8	8.29
Madina	13.52	− 15.25	177	7.24
Kassena Nankana District	10.76	− 1.44	418	6.71
Kenyawegi	− 0.92	34.67	259.9	8.69
Yakepa, close (< 3 km)	7.56	− 8.55	3.65	7.87
Ambodifotatra & Lonkintsy	− 16.98	49.86	92	8.05
Bamako, Sotuba sub	12.65	− 7.93	3.59	7.60
Matola	− 25.95	32.45	52.85	6.46
Lagos, Lemu suburb	6.47	3.37	48	8.01
Barkedji	15.28	− 14.87	114	6.15
Kpetema	8.13	− 11.5	240.9	8.67
Asar	13.75	35.25	0.59	5.89
Kasiga	− 4.82	38.23	620.5	8.00

## Discussion and conclusion

Climate variables play an important role in the dynamics, distribution, and transmission of vector-borne diseases such as malaria. Although rainfall is critical in providing suitable habitats for mosquitoes to breed, it explains little additional variance in malaria transmission; and therefore temperature is a key driver that affects the essential processes of mosquito biology and parasite life cycle [13]. To be more specific, temperature determines transmission intensity, including mosquito development rate, biting rate, and survival of the parasite within the mosquito [11, 43]. Because of the compatibility and relevance of including temperature data into dynamical mathematical models, the study here only accounted for temperature as the main climatic variable.

Mathematical models are generally an abstraction of reality. They have the ability to address the problem in form of equations that capture important linkages of complex transmission dynamics of the disease, which cannot be unraveled using laboratory and ecological experiments [44]. The basic reproduction number *R*_0_ determines the threshold values for which, disease models exhibit changes in their stabilities [45]. The analyses presented in [45] highlighted that, *R*_0_ can provide a reasonable estimate of the reduction level of malaria transmission intensity and therefore it offers a good measure to use as proxy for understanding and analyzing possible options to eliminate the disease [45]. However, the transition from malaria-free to an endemic area is generally associated to different types of bifurcations. Bifurcation arises when a slight increase/decrease in the magnitude of model parameter values triggers a sudden topological change in the disease trend. Traditional mathematical models often consider one endemic equilibrium when *R*_0_ > 1, which is translated to a stable disease-free equilibrium for *R*_0_ < 1 and unstable when *R*_0_ > 1 [46]. In such context, the bifurcation leading from a disease-free equilibrium to an endemic equilibrium is considered forward. Backward bifurcation occurs when the condition *R*_0_ < 1 cannot be used alone to explain all the required disease elimination efforts. In such case, a backward bifurcation point should be identified and the thresholds of the values of *R*_0_ defined for the effective control of the disease. A backward bifurcation point co-exists with the stable disease-free equilibrium [46–48]. Such phenomenon has important public health implications because the condition of having *R*_0_ below unity will no longer be sufficient to guarantee the disease elimination; hence a new threshold for the basic reproduction number should be estimated and considered as the critical point, in which the elimination of the disease can be possible [47, 48]. The hypothesis of making *m*_12_ = *α m*_21_ helped to estimate the magnitude of the threshold (*α*) whose slight increase in the magnitude of its estimated value produces a decrease of *R*_0_ and hence create the possibility to obtain a globally stable endemic area of malaria. In contrast, the disease persists endemically in all cases when *R*_0_ > 1. In the process of using *R*_0_ to estimate the spatial representation of malaria transmission areas, an increase in the difference of *R*_0_(*ΔR*) does not necessarily indicate that the areas in the map are an endemic zone of the disease. In fact, changes of the state of malaria transmission could be noticed for values of *R*_0_ less than unity.

Temperature was used to parameterize the expression of *R*_0_ in patches environment for understanding the dynamics of malaria transmission and later generate maps for the disease risk areas in Africa. Biting rate, vector competence, adult mortality rate, parasite development rate, eggs laid per adult female per day, egg-to-adult survival probability, and mosquito development rate were expressed as dependent on temperature, hence the parameters help to include a climatic variable into the expression of *R*_0_ [13]. Combining these thermal responses constrained from high to low limits into a spatial distribution of temperature values yielded maps showing areas of potential transmission of malaria. The obtained maps of *R*_0_ agree to a certain level with existing malaria risk maps of Africa obtained from an experimental study such as the MARA maps [42]. The present results further agree with the spatial distribution map of *P. falciparum* malaria endemic areas in Africa [49]. The model predicted numerous regions with *R*_0_ greater than unity because of the clumping effects of spatial models [50]. The accuracy of our model is enshrined in the capacity of reproducing almost all known regions at risk of malaria, which were the same as that identified by various other models [42]. The estimates of the basic reproduction number magnitudes for randomly selected points in Africa compared to *APfEIR* further confirmed the accuracy of the approach used. But the variations between *APfEIR* and the values of *R*_0_ in endemic areas were substantial due to the short-range heterogeneity used in the computation of *R*_0_ compared to the patchy distribution of the field datasets. Usually, *APfEIR* are computed with data obtained from disparate health units within the region to characterize the point location, whereas the values of *R*_0_ is estimated based on the exact values of the temperature at point location represented by its geographical coordinates.

In literature, several studies have used different approaches to tackle problems similar to the current study. For instance, Craig et al. [42] have proposed a “fuzzy logic” model for the distribution of stable malaria transmission in sub-Saharan Africa. However, the model development required detailed information, not always available in all localities of Africa. The study in [49] interpolated the probabilities of *P*. *falciparum* entomological inoculation rate (*PfEIR*), *P*. *falciparum* basic reproductive number (*PfR*), and *P*. *falciparum* parasite rate (*PfPR*) to generate the map for each quantity, which were then combined to yield the malaria transmission map. Although the outcome was realistic, the method did not include the dynamics of malaria vectors and parasites. Other studies [28, 29] used CLIMEX model, a platform designed to infer species responses to environmental conditions and climatic parameters to produce potential distributional maps of disease occurrence. The authors only focused on *A. gambiae* and *A. arabiensis* distributions to infer malaria transmission, with no consideration of the parasites. Overall, malaria parasite transmission intensity is spatially heterogeneous and this heterogeneity has important implications for risks and age patterns of progression from malaria infection to disease, infirmity, morbidity, and death [51]. The present study is among the very first investigations that define the limits of contemporary malaria transmission, using the basic reproduction number derived from a dynamical mathematical model. The authors in [14] used a similar approach but limited the study to expected changes in transmission at the Republic of Tanzania. In addition, the models used by Parham and Michael [14] failed to predict accurately the optimal value of temperature for malaria transmission, thus producing maps with false high magnitude areas of malaria transmission. Studies using approaches similar to what is presented here were applied to map bluetongue virus in the Netherlands [52]; to develop a temperature-driven map of the emerging tick vector of Lyme disease *Ixodes scapularis* in Canada [53], and to assess the effect of climate change in the risk of Chagas disease transmission in Colombia [54].

The link between climate change and vector-borne diseases such as malaria is well established [55]. Although other studies highlighted the importance of climate change on the burden of malaria in Africa [56, 57], few researches have considered temperature as a paramount climatic factor and used it to predict future changes in malaria transmission. Our findings showed that changes in the magnitude of temperatures are likely to create a shift in the distribution of malaria-endemic areas than to expand its geographic ranges. This finding concurs with the results reported in [57]. A similar observation was also reported in studies by [28, 29]. It is important to note that, in cases where temperature extremes set boundaries on the vectors distributions, climate change might alter the range (in altitude or latitude) of favorable environmental conditions for the malaria vectors and the parasites. We noted that the greatest effect of climate change on malaria is likely to be observed at a temperature equal to 25 °C, corresponding to the favorable conditions for disease transmission. The optimum temperature value for malaria transmission has been estimated based on the vectors and parasites biological characteristics. With species evolution and under certain circumstances, the vectors and parasites could undergo some genetic modifications in order to adapt to current environments with different optimum temperatures. With this hypothesis, the range for the shift of malaria transmission/endemic areas will be less than predicted. In addition to the direct influence of temperature on the biology of vectors and parasites, changing precipitation patterns could also have short- and long-term effects on vector development [17]. A recent analysis of global mean surface precipitation over the period 1901–1995 indicates that precipitation trends vary across Africa. Precipitation appears to be increasing in east Africa but decreasing in western and northern part of the continent [17]. These are common observations that if actualized, may reduce the level of certainty on our findings.

The study reported in [20] assessed the contribution of different malaria interventions and revealed a considerable fall in malaria burden in sub-Saharan Africa beginning to the year 1980. The decline of malaria burden is partly due to a number of vector control tools [insecticide-treated nets (ITNs), indoor/aerial spraying and other], which have been developed and extensively used. Improvement in diagnosis and treatment has also contributed to the decline of the malaria burden. However, the hypothesis supporting the introduction of interventions here emphasized more on the vector control aspect, which is justified by selecting model parameters (mosquito biting rate, vector competence, adult mosquito mortality rate, and the probability that mosquito eggs survive to become adult) that directly affect malaria vectors for manipulations. Depending on the disease ecology and the policies of the government in each country, the control of malaria often uses multiple intervention measures packaged in an integrated vector management strategy. With the developed model, it was found that if the rate of interventions continues as present, a considerable reduction of malaria transmission is likely to happen by the year 2050 in Africa. However, focusing at an individual country such as Cameroon, in which the latest report is dated 2004 [41]; our findings only revealed a little decline in the prevalence of malaria. Such an outcome may be attributed to the lack of adoption and failure to implement the Abuja plan of action [40, 41] and the low number of projects and research activities directed towards reducing malaria burden as compared to other regions of Africa. On the contrary the application of intervention measures has substantially contributed to the reduction of the burden of malaria in several areas of Kenya, with up to 70% decline in malaria morbidity. An identical projection was obtained by the current study. Moreover, it is important to acknowledge that the existence of diverse malaria environments in Africa, each requiring focal intervention packages to achieve success in disease control. Herein, interventions were applied identically for each country creating a possibility of bias in the overall results.

In summary, by applying the basic reproduction number *R*_0_ derived from a dynamical model in patches environment, the map of malaria transmission intensity was obtained. The findings in this research could constitute a realistic basis for understanding the interactions and complexities between the disease (malaria), its vectors and the parasites. Including interventions in the analysis allowed to measure the level at which, continued efforts made by different governments in Africa have so far contributed to the reduction of the malaria burden. Considerable, complementary and concurrent efforts are still needed in the drive towards malaria eradication, especially in the context of climate change.
